# Authors are also reviewers: problems in assigning cause for missing negative studies

**DOI:** 10.12688/f1000research.2-17.v1

**Published:** 2013-01-21

**Authors:** Stephen Senn

**Affiliations:** 1 Competence Center for Methodology and Statistics, Centre de Recherches Sante, Strassen, Luxembourg, Luxembourg

## Abstract

I compare two possible extreme hypotheses regarding submission of papers to journals: the Q hypothesis, whereby the decision to submit is based on quality of research; and the P hypothesis, whereby it is based on probability of acceptance. I give five reasons as to why the P hypothesis is more plausible and suggest that problems of missing data may previously have caused researchers to misinterpret the evidence on editorial bias.

## Communication

Iain Chalmers and Kay Dickersin have written an interesting commentary ^1^ in this journal on an earlier paper of mine ^2^. I am grateful for the attention they have paid to my article but do not agree with their conclusions for reasons I set out below.

A key characteristic of work from the evidence based medicine (EBM) movement has been its stress on the dangers of bias ^3^ and the acclamation of the randomised clinical trial (or sometimes the meta-analysis of such) as representing the very highest level of evidence ^4^. However, EBM enthusiasts sometimes forget that the same pitfalls that beset observational studies of the effects of treatment are a danger for observational studies of the *process* of evaluating evidence. The claim I made in my previous paper in this journal ^2^ was that researchers in the field of *evidence methodology* had failed to appreciate the problems with the research instrument they were using and that, in consequence, this research was fundamentally flawed. Much of the argument presented by Chalmers and Dickersin in their commentary on that paper ^1^ consists of citing the research I had already called into question, for example the 2002 *JAMA* paper ^5^. I think the research was flawed whereas, presumably, they do not but, whatever their opinion, simply citing such research does not answer my criticisms.

We can consider two extreme hypotheses: the *Q hypothesis* and the *P hypothesis* (Mixtures of these two extreme cases can be envisaged, but to understand the problem it is sufficient to consider the extremes.). The Q hypothesis is necessary if the sort of research that Chalmers and Dickersin ^1^ cite is to be valid. The Q hypothesis supposes that negative and positive studies submitted to journals are comparable in terms of *quality*. That being so, a difference in acceptance rates for negative and positive studies would be evidence of editorial bias, and the fact that such a difference is not found is reassuring. On the other hand, the P hypothesis supposes that a rational decision to submit to a journal would be based on *probability* of acceptance, which cannot thus (necessarily) be expected to differ by outcome of study, even when bias is present. Thus, an editorial bias would be shown by difference in *quality* of accepted negative and positive studies *despite* equal probabilities of acceptance. Equality in acceptance rates would only be reassuring as regard lack of editorial bias if quality did not differ.

The P hypothesis involves a sort of reverse causation: perceived probability of a *future* event is what triggers submission and this determines the quality of what is submitted. If the P hypothesis is correct, then EBM researchers who followed the Q hypothesis, which (implicitly) was the case in the *JAMA* paper that Chalmers and Dickersin cite ^5^, have got things back to front. This may seem far-fetched, but it would not be the first time that such a mistake has been made. For example, some years ago a study showed that Academy Award (‘Oscar’) winners lived longer than a control group of non-winning actors ^6^. This was interpreted as showing the benefit of esteem in terms of years of life gained. However, a more careful analysis suggested it was long life that increased your chance of winning and not *vice versa* ^7^. I used to explain the point at issue to my students thus: to discover that those who had ever received telegrams from the (British) Queen were unusually long-lived (you would not be proof of the life-preserving effect of royal telegrams, since you receive one if you live to be one hundred.

Another example can be given. The TARGET study compared lumiracoxib to naproxen and ibuprofen in more than 18,000 patients suffering from osteoarthritis. Patients were stratified by concomitant low-dose aspirin use ^8, 9^. An interesting finding was that aspirin users had a significantly higher rate of cardiovascular events than non-aspirin users. The authors commented that this was ‘as expected’ ^8^ (p679). In view of the considerable experimental evidence on the cardiovascular prophylactic efficacy of aspirin ^10^, why did they expect this result and not regard it as paradoxical? The answer is that they took it as *obvious* that anticipated cardiovascular events would increase the probability of low-dose aspirin usage and allowed for it in the design. This reverse-causation explanation *reconciles the experimental and the observational evidence*.

In short, the way in which the data have arisen needs to be considered carefully and failure to do so is a fault of many of the studies that Chalmers and Dickersin or, for that matter, Goldacre ^11^ cite. A further irony is that the whole reason why missing negative studies are of such concern is that their missingness causes a bias in evaluating the effects of treatment. The authors of the paper ^5^ cited by Chalmers and Dickersin failed to notice what they should have been sensitised to spot: the studies’ missingness also causes a bias in evaluating the editorial process. The central issue is, ‘what would happen to the studies authors *don’t* submit if they *did* submit them?’ It is naïve to suppose that a *simple* comparison of studies they *do* submit can say what would happen to those they *don’t*. Since my investigation of this issue was inspired by reading *Bad Pharma* ^11^, I can’t resist putting it like this: neither the US Food and Drug Agency (FDA) nor the European Medicines Agency (EMA) accept as a ‘strategy’ for dealing with missing data, ‘just ignore the problem and analyse as usual’ ^12– 14^.

In fact, I consider the P hypothesis is more reasonable that the Q hypothesis for five reasons, some theoretical and some empirical. I list them below.

1. If researchers behave rationally they will submit according to perceived probability of acceptance. We can suppose that there is a reward, *R*( *Y*), and a cost *C*( *Y*) of submission of a study with outcome *Y* where *Y* = 0,1 according to whether the study is negative or positive. The expected return of submission is positive if *P*(q, *Y*) *R*( *Y*) – *C*( *Y*) > 0, ∴ *P*(q, *Y*) > *C*( *Y*)/ *R*( *Y*), where *P*(q, *Y*) is the probability of acceptance seen as an increasing function of the quality, q, of the study and also (possibly, for this is the point to be examined) of the outcome. If *C*(1)/ *R*(1) = *C*(0)/ *R*(0), which is to say that if the ratio of cost to reward is independent of outcome, then the threshold probability at which authors will submit to a journal is identical for both negative and positive studies, without any implication that the quality will therefore be the same. If *C*(1)/ *R*(1) < *C*(0)/ *R*(0) then the threshold probability of acceptance would actually have to be higher for negative than positive studies. Under neither case would observed equal acceptance rates be a proof of lack of editorial bias.

2. As Chalmers and Dickerson note, there is considerable *experimental* evidence that reviewers are more likely to reject negative versions of a given study. Under the P hypothesis, this is easily reconciled with the observed equality of rejection rates in *observational* studies. Under the Q hypothesis, the observational and experimental results are at variance with each other. Thus, just as the reverse-causation hypothesis reconciles experimental and observational data on aspirin, so does the P hypothesis for editorial bias.

3. As Chalmers and Dickersin note, we have evidence that authors are less likely to submit negative studies than positive ones. This makes it improbable that the mixture of studies by quality submitted to journals will be identical, which is what the Q hypothesis requires. However, the P hypothesis does not require quality to be equal between submitted positive and negative studies.

4. In support of this we have observational evidence that the quality of submitted negative studies is higher than positive studies despite acceptance rates being the same ^15^. This is exactly what the P hypothesis predicts, but is not compatible with the Q hypothesis.

5. However, the most important point is one everybody seems to have overlooked. By and large authors and reviewers are (in the long run) the same. I doubt that the experience of Chalmers and Dickerson is much different from my own. I write a lot and I review even more. I have a rule of doing one review (if asked) and no more for journals I have no intention of writing for, but review regularly for those journals I publish in often (for example, *Statistics in Medicine*). Thus I review (mainly) for what I write in, although as a medical statistician I probably review more papers by physicians than physicians do by me. It is true that in his extensive analysis of editorial boards of journals in information science ^16^, Cabanac found the hardly surprising result that editorial board members had in general some considerable seniority whether measured in years since first published or number of published papers, and one might expect that very junior researchers are more likely to submit papers than review them. However, it is pretty obvious that most researchers do both. In their proposal for improving peer review, Hauser and Fehr took it as being so obvious that referees were authors that they suggested punishing tardy reviews by placing the reviewer’s next paper as author in a ‘sin bin’ ^17^. Thus, reviewers are (mainly) just authors on another occasion. The Q theory requires researchers to have Jekyll and Hyde personalities. Vile hypothesis tester Mr Hyde chooses inappropriately that the negative studies he has conducted should not be submitted, while journal reviewer Dr Jekyll justly judges similar studies with the Wisdom of Solomon. Faced with a negative paper the referee asks, ‘would I submit something like this?,’ answers, ‘No!,’ and then recommends publication. I regard this as improbable. This leads to my main point.

My main point picks up on my fifth reason. The whole business of what gets published and what does not does not lend itself to separation. This is a point I made in my original paper. Chalmers and Dickersin ^1^ dismiss this, but I stand by my original statement. The studies that the EBM movement has carried out *fail by the very standards the movement promotes elsewhere*. Fairness applies not only to the business of judging the effects of medicines but to the business of judging the business by which they are judged.

However, I will permit myself an unfair opinion. Nothing much can be hoped for from the sad and sorry mess that is the medical press. I regard it as irredeemable. It makes no difference what the origin of the problem is: whether medical researchers as authors or medical researchers as reviewers are saints or sinners. If they are not guilty one way, they are guilty the other, but the simplest explanation of the facts is that they are guilty in both. In any case, the problem is not just with what is absent, but with what is present. We need to make it possible to check what is published ^18^ and currently very few medical journals do so.

We need to find a radically different solution: one which renders meaningless the accolade of publishing in a ‘leading’ journal, one which shows the impact factor for the fraud it is. We need to make such journals irrelevant for disseminating the results of primary research. We have to look elsewhere for that.
