# Supplementary material for: Authors are also reviewers: problems in assigning cause for missing negative studies
Source: F1000Res. 2013 Jan 21;2:17. [Version 1] doi: 10.12688/f1000research.2-17.v1 (PMC3752705; doi:10.12688/f1000research.2-17.v1)
Supplement: Peer review history

# Peer review: Referee response for version 1

**Published:** 2013-01-29

**Authors:** Sara Hughes

**Affiliations:** VP Head of Clinical Statistics, GlaxoSmithKline (GSK), Uxbridge, Middlesex, UK

In his concluding statement, Senn argues for a radically different solution for disseminating the results of primary research. Expanding his conclusions to add suggestions for how this may be addressed would be valuable.

I have read this submission. I believe that I have an appropriate level of expertise to confirm that it is of an acceptable scientific standard.

# Peer review: Referee response for version 1

**Published:** 2013-01-28

**Authors:** James Matcham

**Affiliations:** Director, Biostatistics, Amgen Ltd, Cambridge, UK

I have read this submission. I believe that I have an appropriate level of expertise to confirm that it is of an acceptable scientific standard.

# Peer review: Referee response for version 1

**Published:** 2013-01-28

**Authors:** Steff Lewis

**Affiliations:** Public Health Sciences, University of Edinburgh Medical School, Edinburgh, UK

I have read this submission. I believe that I have an appropriate level of expertise to confirm that it is of an acceptable scientific standard.

# Peer review: Referee response for version 1

**Published:** 2013-01-22

**Authors:** Paul Silcocks

**Affiliations:** Cancer Research UK Ltd, University of Liverpool, Liverpool, UK

I find Senn’s arguments both plausible and persuasively expounded. I am surprised that the potential confounding by study quality of the relation between acceptance rates and type of outcome has not been more widely studied, although I, like Chalmers and Dickens, am puzzled by Senn’s statement ^1^ that *“it also seems plausible that higher quality studies are more likely to lead to a positive result”* – possibly what is intended here is *“more likely to lead to a convincing result either way”*.  The correspondence as a whole would be strengthened by distinguishing truly  “negative” results from those that are merely inconclusive.

I have read this submission. I believe that I have an appropriate level of expertise to confirm that it is of an acceptable scientific standard.

## References

[1] SennS: Misunderstanding publication bias: editors are not blameless after all [v1; ref status: indexed, http://f1000r.es/YvAwwD]. * F1000Research*.2012;1(59) : 1710.12688/f1000research.1-59.v1PMC378236524358828
